# Long non-coding RNA AK027294 involves in the process of proliferation, migration, and apoptosis of colorectal cancer cells

**DOI:** 10.1007/s13277-015-4350-x

**Published:** 2016-01-28

**Authors:** Hui Niu, Zhaoyang Hu, Hui Liu, Guoliang Hu, Bo Yang, Shixiu Wu, Fang Li

**Affiliations:** 1 Academy of Military Medical Sciences, Beijing, 100850 China; 2 Tumor Research Institute, Hangzhou Cancer Hospital, Hangzhou, 310002 China; 3 Department of Oncology, Hainan Branch of PLA General Hospital, Sanya, 572013 China; 4 Third Healthcare Division, Hainan Branch of Chinese PLA General Hospital, Sanya, 572013 China; 5 Department of Oncology, General Hospital of Chinese PLA, Beijing, 100853 China

**Keywords:** Colorectal cancer, Long non-coding RNAs, AK027294, Proliferation, Migration, Apoptosis

## Abstract

This study is aimed to investigate the differentially expressed long non-coding RNAs (lncRNAs) in colorectal cancer and its potential biological function. Colorectal adenoma is the precancerous lesions of colorectal cancer, so in this study, we used colorectal adenoma as negative control. The global lncRNA expression profile in colorectal cancer and adenoma was evaluated by bioinformatics. The biological functions and potential mechanism of AK027294 were investigated in HCT116, HCT8, and SW480 colorectal cancer cells. A total of 135 lncRNAs were found to be differentially expressed in colorectal cancer and adenoma tissues. Among them, 71 lncRNAs were up-regulated and 64 lncRNAs were down-regulated. Especially, AK027294 was found to be highly expressed in colorectal cancer tissues compared with colorectal adenoma tissues (fold change is 184.5). Our results indicated that AK027294 down-regulation significantly inhibited colorectal cancer cells proliferation and migration, but promoted cell apoptosis (*P* < 0.05). The potential mechanism of AK027294 might be associated with the regulation of caspase-3, caspase-8, Bcl-2, MMP12, MMP9, and TWIST. The lncRNA expression profile in colorectal cancer suggests lncRNAs may play important roles in the occurrence and progression of colorectal cancer. AK027294 is highly expressed in colorectal cancer and closely correlates with colorectal cells proliferation, migration, and apoptosis.

**Electronic supplementary material:**

The online version of this article (doi:10.1007/s13277-015-4350-x) contains supplementary material, which is available to authorized users.

## Introduction

Colorectal cancer (CRC) is the third most common cancer in the world, with an estimated incidence of more than one million case [1, 2]. Although the long-term survival rates for patients with early CRC have been improved based on the improvement of diagnosis and treatment, the prognosis of patients with advanced disease remains unsatisfied [3]. Therefore, some specific biomarkers associated with CRC will be potentially helpful for the prognosis of these patients.

Long non-coding RNAs (lncRNAs), sized from 200 nt to 100 kb, is reported to be vital for governing fundamental biological processes, such as cell proliferation, cell migration, cell cycle, and cell apoptosis [4–6]. However, primary studies reported that lncRNAs were transcriptional “noise” without biological functions [7, 8]. Recently, lncRNAs have been reported to be associated with tumor formation and progression [9, 10]. For example, Chung et al. reported that prostate cancer non-coding RNA 1 (PRNCR1) was involved in the occurrence of prostate cancer [11]. Gao et al. demonstrated that LncRNA-HOST2 played a key role in regulating cell biological behaviors in ovarian cancer [12]. In colorectal cancer, novel lncRNA RP11-462C24.1 was found to be lowly expressed and correlated with patients’ prognosis [13]. Moreover, Han et al. screened lymph nodes metastasis associated long noncoding RNAs (lncRNAs) by microarray analysis and found that 545 lncRNAs were differentially expressed in metastatic lymph node in comparison with normal lymph node (NLN) [14]. However, potential lncRNAs associated with colorectal cancer has never been reported.

In this study, firstly we explored the differentially expressed lncRNAs in colorectal cancer tissue compared with colorectal adenoma tissues by using bioinformatics, then we screened out the most differentially expressed lncRNAs with biggest fold change. After this, we further detected the biological functions and potential mechanism of the screen out lncRNAs, for instance, the effects of the screen out lncRNAs on the proliferation, migration and apoptosis of colorectal adenoma cells. It may provide a promising target gene to prevent the development and progression of colorectal cancer.

## Materials and methods

### Bioinformatics analysis

We obtained the GSE37364 microarray date from PUBMED GEO Datasets (http://www.ncbi.nlm.nih.gov/geo/query/acc.cgi?acc=GSE37364). GSE37360 microarray information: total RNA was extracted from colonic biopsy samples of histologically negative patients and of patients with adenoma or colorectal cancer and were hybridized on Affymetrix HGU133 Plus 2.0 microarrays. Then 5635 lncRNAs gene expression data were obtained (Supplementary Data 1). The whole genomic microarray analysis was performed in order to identify gene expression profile alterations focusing on the dysplastic adenoma-carcinoma transition. In this study, we used this microarray data to screen out the differentially expressed lncRNAs.

### Patients information

In this study, three colorectal cancer tissues and one colorectal adenoma tissue were obtained from Hai Nan Branch of PLA General Hospital, and tqo colorectal adenoma tissues were obtained from Hangzhou cancer hospital. Colorectal cancer tissues were from two males and one female with mean age of 54 years. Adenoma biopsy tissues were from two males and one female with mean age of 55 years.

### RT-PCR

Total RNA was extracted from cultured HCT116 cells or patients’ adenoma and tumor tissues using TRIzol (Invitrogen Life Technologies) and determined its concentration was determined. Total RNA (0.5 μg) was used as a template to prepare cDNA (Reverse Transcription System, Promega Corporation, Madison, WI, USA; cat. no. A3500). The messenger RNA (mRNA) expression of target genes was quantified using SYBR Premix EX Taq (Takara Bio, Inc., Shanghai, China) on the ABI 7500 squence detection system (Advanced Biosystem, Thermo Fisher Scientific, Waltham, MA, USA). PCR was performed with the following thermocycling conditions: an initial of 5 min at 95 °C, followed by 40 cycles of 95 °C for 30 s, 55 °C for 30 s, and 72 °C for 30 s. The thermocycler used in the present study was the StepOnePlus™ Real-Time PCR system (Applied Biosystems Life Technologies, Foster City, CA, USA). All experiments were performed in triplicate. Finally, the 2‑ΔΔCt method was performed to calculate the relative expression. The primers were obtained from Funengbio Co. (Shanghai, China). The primer sequences are as follows.

**Table:** 

Name	Forward	Reverse
AK027294	ATGACACCTATTGGAGAA	TAAGCACACCTGAGTAAT
AK001058	GAAGCAGAGTTGTTGATT	ATAGAGATATGTATCCAGTGT
AK025818	CTTTATTTAGTCTGTTGCCTCTG	GATGTGCTGCTCTGATGT
AK094088	CACTTATCACTACCTGTTG	GTTCTGCCACTTAATAGC
BC137325	TACTCTTGGTTGTCTTCCTA	GCTGTCTCTTGTGATGAAT
CR749831	CTCTGGACTGATACAATAAGC	TTCTGGACCTCTAACTAACC
AK000106	AAGTAGCAACCAGATGTG	AAGGATGTGAAGTAGTCATT
AB002438	GCTTGGAGAGGTAATAAC	TATGCTTGAGGATAGGAA
AJ420553	TGTTCTCTGCTTGCTACC	CTCCCACTGTTGCCTATT
AK023519	CTTCACTGTTGTTCACTTCATAA	GATTCTCATAAGGCTAAGGACTAT
U6	ATTGGAACGATACAGAGAAGATTA	AATATGGAACGCTTCACGAAT
TWIST1	TTCTCGGTCTGGAGGATGGA	CCACGCCCTGTTTCTTTGAAT

### Cell culture

Human CRC cell lines HCT116, HCT8, and SW480 was acquired from the American Type Culture Collection (ATCC) and cultured in Dulbecco’s modified Eagle’s medium (DMEM) supplemented with 5 % fetal bovine serum (FBS) in a humidified incubator with 5 % CO_2_ atmosphere at 37 °C.

### RNA interference

HCT116 cells were transiently transfected with negative control small interfering RNA siRNA (200 nM, target gene: caatgtatacattatggcag) and AK027294 siRNA (200 nM) (siRNA1 target gene: caatggtgtggcctgattc; siRNA2 target gene: agcagcttattctaccag; siRNA3 target gene: agacctgaatgacacctat), respectively, using lipofectamine 2000 reagent (Invitrogen, Carlsbad, CA, USA) according to the manufacturer’s instructions. These siRNAs were commercially synthesized by Funengbio Co. (Shanghai, China). After incubated for 24 h, cells were collected for follow experiments.

### Wound healing assays

HCT116 cells were cultured in 6-well plates and transfected with different oligonucleotides. When growing to about confluent of 90 %, cells were scratched by a sterile 10 μl pipette tip and incubated in a humidified incubator with 5 % CO_2_ atmosphere at 37 °C. Then the open wound area was measured at 0 and 24 h. The experiments were repeated for three times.

### Transwell invasion assay

After transfection at 24 h, cells (2 × 10^5^) in 100 μl of serum-free DMEM were cultured in 8-μm culture inserts. Then, the lower chamber of the culture inserts was filled with 20 % FBS-DMEM. Cells were cultured for 24 h, fixed with methanol for 30 min, and stained with hematoxylin for 20 min. Finally, migrated cells were counted in five random fields under an inverted microscope (×20).

### CCK-8 assay

About 2 × 10^3^ cells were plated in 96-well plates. Cell proliferation was evaluated using Cell Counting Kit-8 (KeyGEN biotech, Nanjing, China) in accordance with the manufacturer’s instructions. The cell proliferation curves were plotted by the absorbance values at different time point. All experiments were performed for three times.

### Western blotting

Total protein was collected using a lysis buffer and quantified by bicinchoninic acid (BCA) method. Cell lysates were separated using 10 % SDS-PAGE and transferred to the polyvinylidene difluoride membrane. The membranes were immersed into PBST solution supplemented with 5 % non-fat milk for 2 h. Then, the membranes were incubated with antibody-caspase 3 (dilution 1:200; Santa Cruz, CA), antibody-caspase 8 (dilution 1:200; Santa Cruz, CA), antibody-Bcl2 (dilution 1:200; Santa Cruz, CA), antibody-TWIST1 (dilution 1:500; Santa Cruz, CA), and antibody-GAPDH,(dilution 1:1000; Santa Cruz, CA), respectively, at 4 °C overnight. All membranes were incubated with appropriate second antibodyat room temperature for 1 h and visualized by enhanced chemiluminescence (PierceBiotechnology, Inc).

### Apoptosis analysis by flow cytometry

Cells (1 × 10^5^) were collected by using Trypsin-EDTA and washed with PBS. Then, cells were centrifugated and incubated with propidium iodide (PI; Sigma, USA) and annexin V-FITC (KeyGEN biotech, Nanjing, China) for 15 min at room temperature. Then, cell apoptosis was assessed by flow cytometry. Each test was repeated three times.

### Statistical analysis

All statistical analyses were performed by using SPSS19.0. Independent-sample *t* test was used to compare the difference between two groups. *P* < 0.05 was considered to be statistically significant. And the significance level was defined as *P < 0.05, **P < 0.01, and ***P < 0.001.

## Results

### LncRNA expression profiles in colorectal cancer and adenoma tissues

**Fig. 1:**
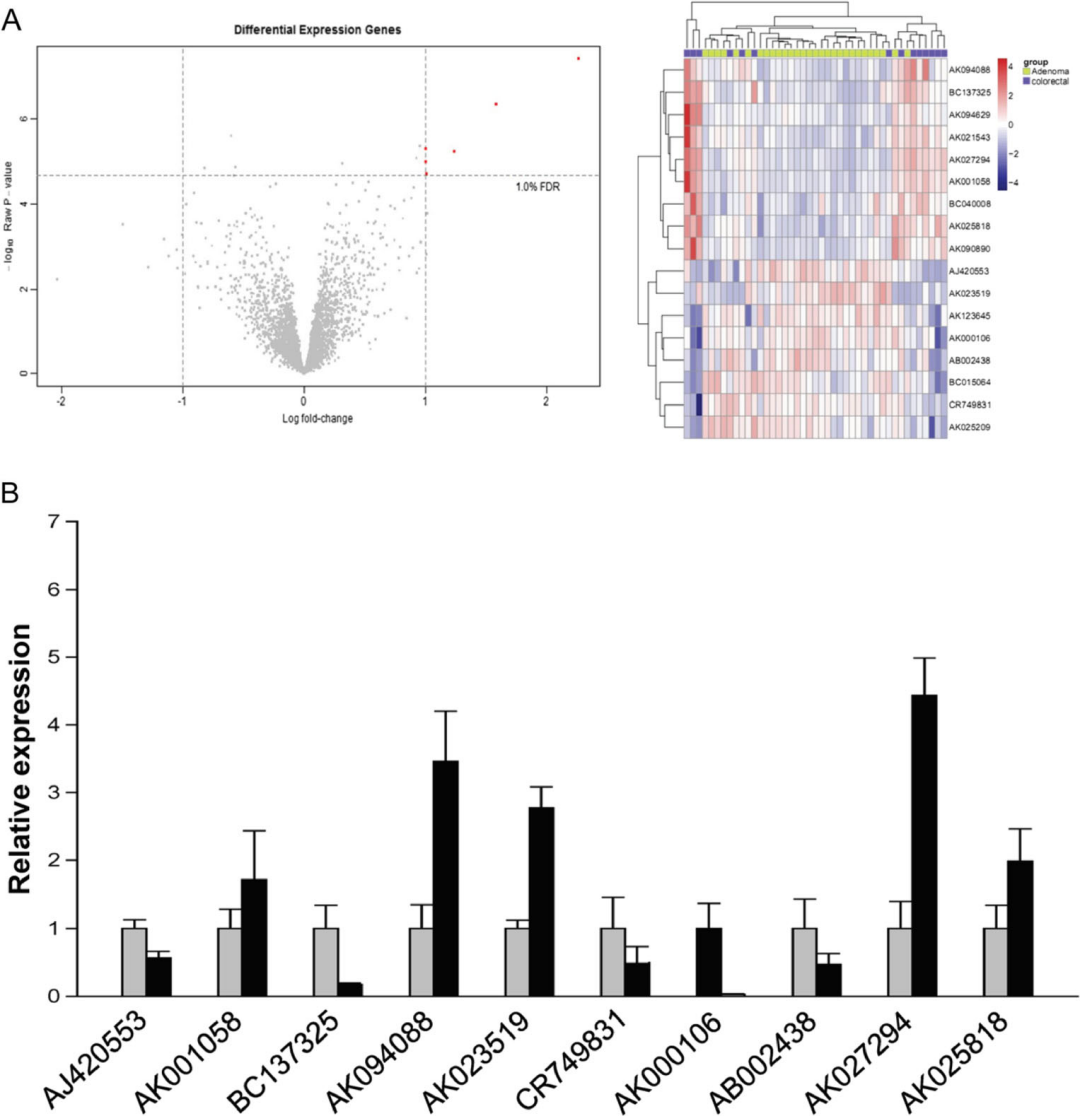
The screening of differentially expressed lncRNAs. **a** GSE37364 microarray data analysis is to identify differentially expressed lncRNAs genes. The volcano plot and heat map shows the differential lncRNAs expression profiles between colorectal cancer and adenoma tissues. *Red* indicates high relative expression, and *Blue* indicates low relative expression. **b** The verification of top 10 differentially expressed lncRNAs in vivo by RT-PCR, including AK027294, AK001058, AK025818, AK094088, BC137325, CR749831, AK000106, AB002438, AJ420553, and AK023519

**Fig. 2:**
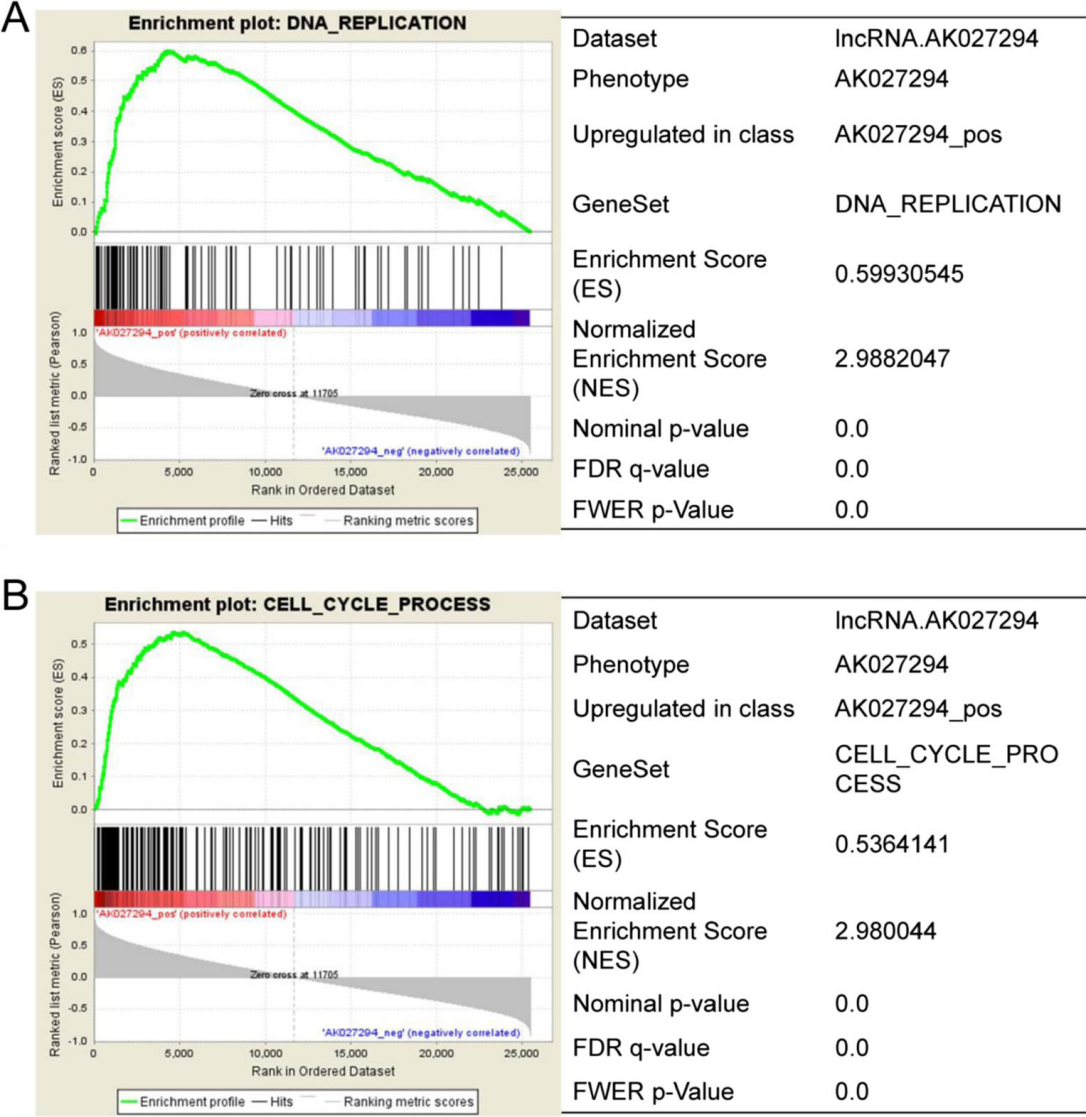
AK027294 correlated with DNA replication (**a**) and cell cycle process (**b**) by enrichment of signaling pathways, the normalized enrichment score (*NES*) for DNA replication and cell cycle process is 2.988 and 2.980, respectively

When the adjusted *P* value was less than 0.01 and the fold change ≥2 in colorectal cancer tissues compared with adenoma tissues, it will be regarded as differentially expressed lncRNAs. A total of 135 lncRNAs were found to be significantly differentially expressed in colorectal cancer tissues compared with adenoma tissues. Among them, 71 lncRNAs were up-regulated and 64 lncRNAs were down-regulated in colorectal cancer (Supplementary data 2). The top 10 differentially expressed lncRNAs in colorectal cancer tissues were AK027294, AK001058, AK025818, AK094088, BC137325, CR749831, AK000106, AB002438, AJ420553, and AK023519, respectively (Fig. 1a; Table 1). Furthermore, the most differentially expressed lncRNA is AK027294 (fold change = 184.5). Then, we verified the expression of these lncRNAs in vivo; the colorectal cancer tissues and colorectal adenoma tissues were obtained from patients and used to extract RNA to perform RT-PCR. As shown in Fig. 1b, although the expression of AK023519 and BC137325 were different with the bioinformatics analysis, the expression of AK027294 was still highest. In addition, according to the enrichment of signaling pathways, we found that AK027294 was correlated with DNA replication and cycle process in colorectal cancer (Table 2; Fig. 2), suggesting that AK027294 might play an important role in biological function of colorectal cancer cells.

**Table 1:** More than twofold differentially expressed lncRNAs in colorectal cancer tissues comparing with colorectal adenoma tissues

Name	Log FC	*P* value	Relative expression	
			Colorectal adenoma	Colorectal cancer
AK027294	2.266403	3.88E−08	5.008716819	7.275120094
AK001058	1.584973	4.62E−07	6.350289182	7.93526256
AK025818	1.239902	6.08E−06	5.768486904	7.008388975
AK094088	1.016936	0.00017	4.068249686	5.085185471
BC137325	1.008726	1.99E−05	4.868172279	5.876898392
CR749831	−1.11336	0.001701	8.834062854	7.720700755
AK000106	−1.15427	0.000707	7.739382039	6.585108512
AB002438	−1.28354	0.003101	7.104724846	5.821187394
AJ420553	−1.49459	0.000309	7.007212845	5.512623827
AK023519	−2.03528	0.005999	6.863707448	4.828429247

**Table 2:** Potential function of AK027294 was analyzed by enrichment of signaling pathways

Name	Size	NES	*P* value
DNA replication	98	2.988205	<0.001
Cell cycle process	189	2.980044	<0.001
Mitotic cell cycle	151	2.929835	<0.001
M-phase of mitotic cell cycle	83	2.90654	<0.001
DNA metabolic process	249	2.899867	<0.001
Cell cycle	310	2.875858	<0.001
Mitosis	80	2.828547	<0.001
Cell cycle phase	166	2.820341	<0.001
M-phase	110	2.812781	<0.001
DNA-dependent replication	54	2.738393	<0.001

### AK027294 down-regulation inhibited the proliferation of colorectal cancer cells

**Fig. 3:**
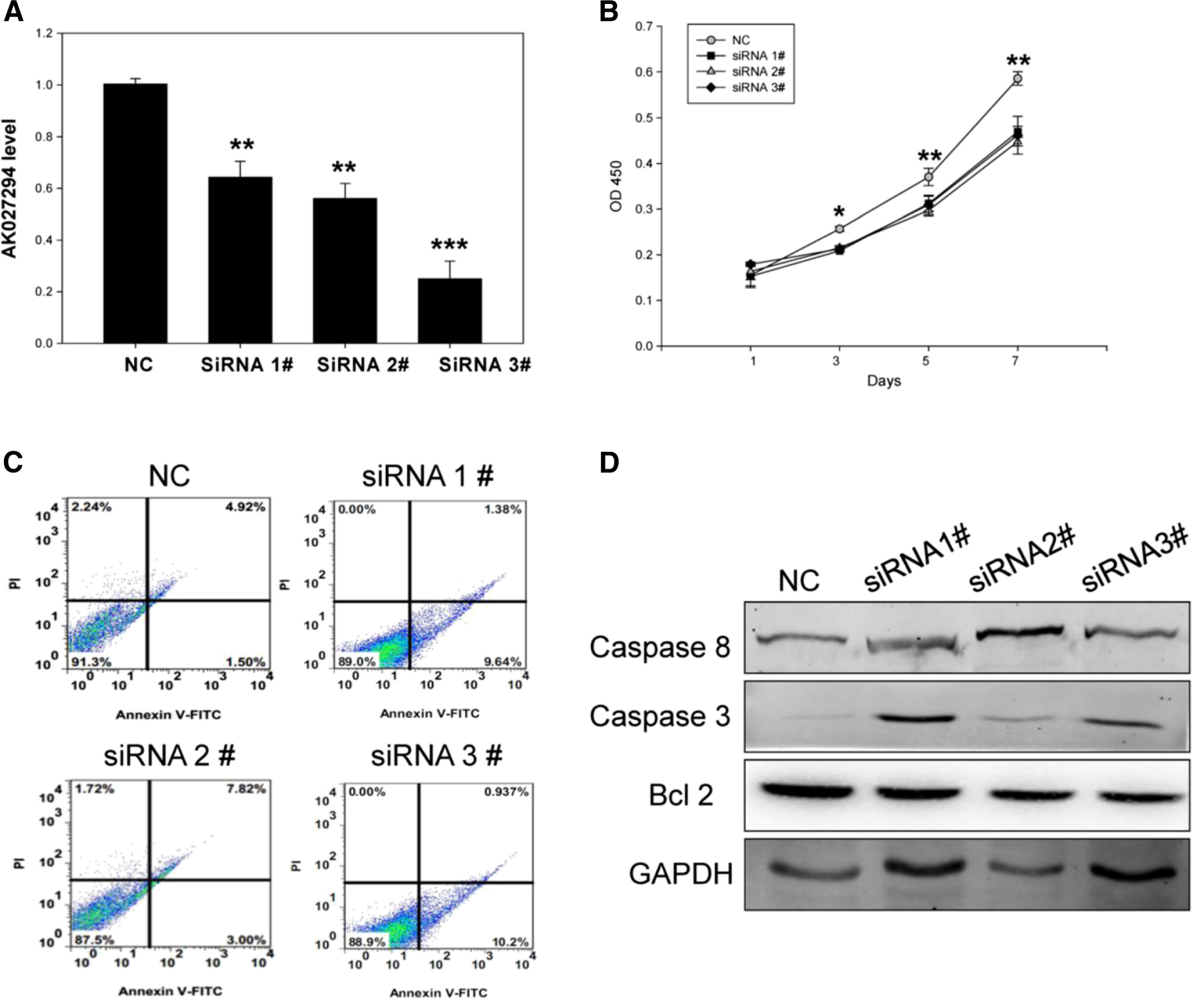
AK027294 down-regulation affects HCT116 cells’ proliferation and apoptosis. **a** AK027294 expression was detected by qRT-PCR. **b** AK027294 down-regulation significantly inhibited cell proliferation by CCK-8 assay. **c** AK027294 down-regulation significantly promoted cell apoptosis by flow cytometry. **d** AK027294 down-regulation affected the expression of caspase 3, caspase 8, and BCl-2. **P < 0.01; ***P < 0.001. *NC* represents negative control

LncRNA microarray assay and gene enrichment analysis suggested that AK027294 was involved in cell cycle process and DNA replication, suggesting that AK027294 might be correlated with cell proliferation and apoptosis. In order to validate the observations, we knocked down the expression of AK027294 in HCT116 colorectal cancer cell line by three specific siRNAs for AK027294. The results were shown in Fig. 3a, indicating different degrees of reduction in the AK027294 expression in AK027294 siRNA-transfected cells (Fig. 3a). CCK-8 assay revealed that when AK027294 expression was down-regulated, the proliferation of HCT116 cells was significantly inhibited compared with negative control siRNA-treated group (Fig. 3b). Furthermore, we also verified this result in HCT8 and SW480 cells. We found that the proliferation of HCT8 cells (Fig. S1a) and SW480 (Fig. S1b) treated with these siRNAs was dramatically decreased than control group. These results were consistent with HCT116 cells’ results, and these results revealed that the down-regulation of AK027294 was able to inhibit the proliferation of colorectal cancer cells.

### AK027294 down-regulation promoted the apoptosis of colorectal cancer cell

Then, we evaluated the effects of AK027294 expression on HCT116 cells’ apoptosis by flow cytometry. As shown in Fig. 3c, when AK027294 expression was knocked down by different siRNAs, cell apoptosis rates (8.96–11.14 %) were markedly increased compared with negative control siRNA-treated group (2.13 %). To explore the potential mechanism of AK027294-regulated HCT116 cells’ apoptosis, we detected the expression of caspase 3, caspase 8, and Bcl-2 by Western blot. The results revealed that the expression of caspase-3 and caspase-8 were enhanced in HCT116 cells with the treatment of AK027294 siRNA compared with control group. In contrast, the expression of Bcl-2 was gradually decreased in HCT116 cells treated with siRNA (Fig. 3d). Moreover, similar results can be seen in HCT8 cells (Fig. S2a) and SW480 cells (Fig. S2b). These results indicated that the down-regulation of AK027294 can promote the apoptosis of colorectal cancer cells.

### AK027294 down-regulation inhibited cell migration and affected the expression of MMP12, MMP9, and TWIST1 in HCT116 colorectal cancer cells

**Fig. 4:**
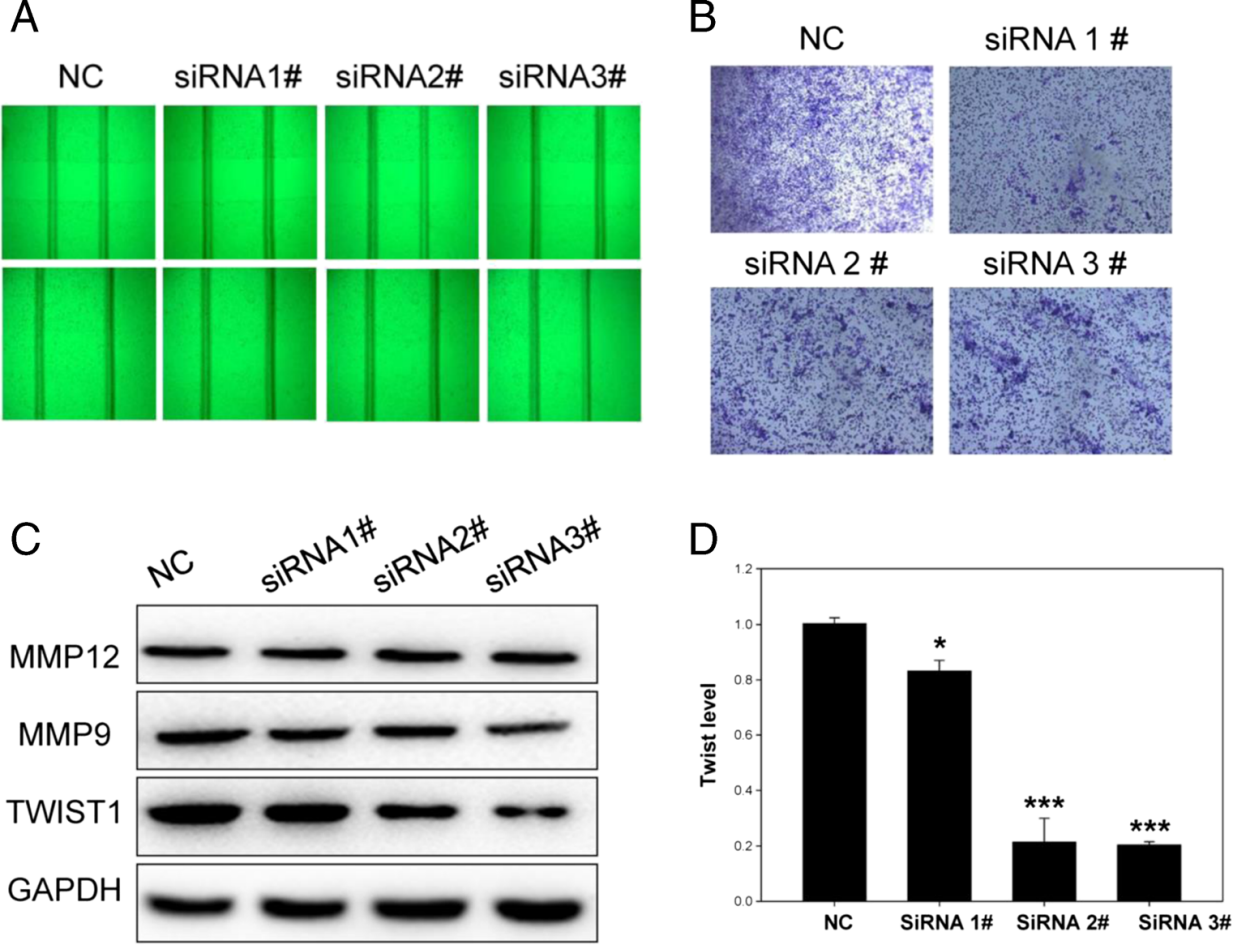
AK027294 down-regulation inhibits cell migration. **a** AK027294 expression inhibited cell migration by wound healing assay. **b** AK027294 expression inhibited cell migration by Transwell assay. **c** Western blot results demonstrated that AK027294 down-regulation induced the decrease of MMP12, MMP9, and TWIST1. **d** QRT-PCR results shown that AK027294 down-regulation resulted in the decrease of TWIST1

In addition, we evaluated the effect of AK027294 on the migration of HCT116 cells by Wound healing assay and transwell assay, respectively. In comparison with negative control siRNA-treated group, wound healing assay exhibited that the ability of cell migration was significantly inhibited when the expression of AK027294 was silenced by the three siRNAs (Fig. 4a). Meanwhile, Transwell assay showed that when the expression of AK027294 was down-regulated, migrated cells were significantly reduced than those in negative control siRNA (Fig. 4b). Moreover, in order to explore the possible mechanism of AK027294 associated with cell migration, we tested the expression of MMP12 and MMP9 in HCT116 cells treated by siRNAs by Western blot. The results demonstrated that AK027294 down-regulation induced the different degree decrease of MMP12 and MMP9 (Fig. 4c). Additionally, the expression of MMP12, MMP9 in HCT8 (Fig. S3a), and SW480 (Fig. S3b) cells was declined when these cells were treated with siRNA. Furthermore, we also detected the expression of TWIST1 by qRT-PCR and Western blot, respectively. Results showed that TWIST1 mRNA and protein levels were apparently down-regulated when AK027294 was knocked down by siRNA2 and siRNA3 than those in control groups (Fig. 4c, d).

## Discussion

Recent studies have demonstrated that lncRNAs are linked to the formation and progression of CRC [13–16]. However, potential lncRNAs related to both colorectal cancer and colorectal adenoma have not been reported. In this study, we screened lncRNA expression profiles in colorectal cancer and adenoma tissues by LncRNA microarray assay. The results revealed that a total of *135* lncRNAs were differently expressed in colorectal cancer and adenoma tissues. It is well known that most colorectal cancers (CRC) arise from colorectal adenomas [17, 18]. Thus, our results revealed that *135* lncRNAs might be potential biomarkers associated with the formation of CRC. According to the data, we found that *71* and *64* lncRNAs were up-regulated and down-regulated in colorectal cancer, respectively. Then, we focused on AK027294 lncRNA, which was found to be correlated with DNA replication and cycle process in colorectal cancer by the enrichment of signaling pathways.

AK027294 lncRNA, a novel molecular, was found to be evidently higher in colorectal cancer than that in colorectal adenoma. Although enrichment of signaling pathways demonstrated that AK027294 was correlated with DNA replication and cycle process, its potential functions remained elusive. Thus, we investigated the biological function of AK027294 in colorectal cancer cells. Results showed that the down-regulation of AK027294 was able to inhibit proliferation and migration of colorectal cells, and promote apoptosis in colorectal cancer. Howerver, the mechanism of AK027294 was little to be known. It is well known the activation of caspase 3 and caspase 8 is responsible for cell apoptosis [19, 20]. Bcl-2, an anti-apoptotic gene, is reported to be correlated with cell apoptosis and proliferation [21, 22]. Thus, we detected the expression of caspase 3, caspase 8, and BCL-2, when AK027294 expression was significantly knocked down in colorectal cancer cells. The current results revealed that down-regulation of AK027294 in colorectal cells induced the increase of caspase 3 and caspase 8 expression but resulted in the decrease of BCL-2 expression in colorectal cancer cells, indicating that AK027294 affected cell apoptosis and proliferation via regulating the expression of caspase 3, caspase 8, and Bcl-2. In addition, it is well known that both MMP9 and MMP12 are involved in the process of cell migration [23, 24]. TWIST1 is frequently reported to be highly expressed in colorectal cancer and is correlated with tumor formation and progression [25, 26]. While the relationships between AK027294 and MMP9, MMP12, and TWIST1 in cell migration remain unknown.

Thus, we detected the expression of MMP9, MMP12, and TWIST1 in colorectal cancer cells. The results showed that MMP9, MMP12, and TWIST1 were down-regulated when AK027294 expression in colorectal cells was knocked down. These results indicated that the effect of AK027294 down-regulation on cell migration was correlated with MMP9, MMP12, and TWIST1.

## Conclusions

In summary, we screen a lncRNA expression profile associated with colorectal cancer and colorectal adenoma. AK027294 is confirmed to be a novel lncRNA, which is correlated with cell proliferation, migration, and apoptosis in colorectal cancer cells. Our findings suggest that AK027294 may play an important role in colorectal occurrence and progression and may be a potential target to prevent progression of colorectal cancer. As this study is the first to report the biological functions of AK027294, further detailed and in-depth investigations are needed to validate our observations.

## Electronic supplementary material

Below is the link to the electronic supplementary material.

ESM 1 (DOCX 130 kb)

ESM 2 (DOCX 160 kb)

ESM 3 (DOCX 139 kb)

ESM 4 (XLS 884 kb)

ESM 5 (XLSX 21 kb)

## References

[1] Wang Z, Sun X, Wang Y (2014). Association between miR-27a genetic variants and susceptibility to colorectal cancer. Diagn Pathol.

[2] Guo Q, Zhao Y, Chen J (2014). BRAF-activated long non-coding RNA contributes to colorectal cancer migration by inducing epithelial-mesenchymal transition. Oncol Lett.

[3] Armaghany T, Wilson JD, Chu Q, Mills G (2012). Genetic alterations in colorectal cancer. Gastrointest Cancer Res.

[4] Han Y, Yang YN, Yuan HH (2014). UCA1, a long non-coding RNA up-regulated in colorectal cancer influences cell proliferation, apoptosis and cell cycle distribution. Pathology.

[5] Hu Y, Chen HY, Yu CY (2014). A long non-coding RNA signature to improve prognosis prediction of colorectal cancer. Oncotarget.

[6] Qi P, Xu MD, Ni SJ (2013). Low expression of LOC285194 is associated with poor prognosis in colorectal cancer. J Transl Med.

[7] Wilusz JE, Sunwoo H, Spector DL (2009). Long noncoding RNAs: functional surprises from the RNA world. Genes Dev.

[8] Geisler S, Coller J (2013). RNA in unexpected places: long non-coding RNA functions in diverse cellular contexts. Nat Rev Mol Cell Biol.

[9] Yang Y, Li H, Hou S (2013). The noncoding RNA expression profile and the effect of lncRNA AK126698 on cisplatin resistance in non-small-cell lung cancer cell. PLoS ONE.

[10] Jiang YJ, Bikle DD (2014). LncRNA profiling reveals new mechanism for VDR protection against skin cancer formation. J Steroid Biochem Mol Biol.

[11] Chung S, Nakagawa H, Uemura M (2011). Association of a novel long non-coding RNA in 8q24 with prostate cancer susceptibility. Cancer Sci.

[12] Gao Y, Meng H, Liu S (2015). LncRNA-HOST2 regulates cell biological behaviors in epithelial ovarian cancer through a mechanism involving microRNA let-7b. Hum Mol Genet.

[13] Shi D, Zheng H, Zhuo C (2014). Low expression of novel lncRNA RP11-462C24.1 suggests a biomarker of poor prognosis in colorectal cancer. Med Oncol.

[14] Han J, Rong LF, Shi CB (2014). Screening of lymph nodes metastasis associated lncRNAs in colorectal cancer patients. World J Gastroenterol.

[15] Zheng HT, Shi DB, Wang YW (2014). High expression of lncRNA MALAT1 suggests a biomarker of poor prognosis in colorectal cancer. Int J Clin Exp Pathol.

[16] Iguchi T, Uchi R, Nambara S (2015). A long noncoding RNA, lncRNA-ATB, is involved in the progression and prognosis of colorectal cancer. Anticancer Res.

[17] Pesson M, Volant A, Uguen A (2014). A gene expression and pre-mRNA splicing signature that marks the adenoma-adenocarcinoma progression in colorectal cancer. PLoS ONE.

[18] Lee TJ, Rees CJ, Blanks RG (2014). Colonoscopic factors associated with adenoma detection in a national colorectal cancer screening program. Endoscopy.

[19] Simpson KL, Cawthorne C, Zhou C (2013). A caspase-3 ‘death-switch’ in colorectal cancer cells for induced and synchronous tumor apoptosis in vitro and in vivo facilitates the development of minimally invasive cell death biomarkers. Cell Death Dis.

[20] Yao Y, Li L, Huang X (2013). SERPINA3K induces apoptosis in human colorectal cancer cells via activating the Fas/FasL/caspase-8 signaling pathway. FEBS J.

[21] Buranabunwong N, Ruangrungsi N, Chansriniyom C (2015). Ethyl acetate extract from Glycosmis parva leaf induces apoptosis and cell-cycle arrest by decreasing expression of COX-2 and altering BCL-2 family gene expression in human colorectal cancer HT-29 cells. Pharm Biol.

[22] Ma L, Li W (2014). Emodin inhibits LOVO colorectal cancer cell proliferation via the regulation of the Bcl-2/Bax ratio and cytochrome. Exp Ther Med.

[23] Jin H, Li XJ, Park MH (2015). FOXM1-mediated downregulation of uPA and MMP9 by 3,3’-diindolylmethane inhibits migration and invasion of human colorectal cancer cells. Oncol Rep.

[24] Anghelina M, Schmeisser A, Krishnan P (2002). Migration of monocytes/macrophages in vitro and in vivo is accompanied by MMP12-dependent tunnel formation and by neovascularization. Cold Spring Harb Symp Quant Biol.

[25] Valdes-Mora F, Gomez del Pulgar T, Bandres E (2009). TWIST1 overexpression is associated with nodal invasion and male sex in primary colorectal cancer. Ann Surg Oncol.

[26] Kim YH, Kim G, Kwon CI (2014). TWIST1 and SNAI1 as markers of poor prognosis in human colorectal cancer are associated with the expression of ALDH1 and TGF-beta1. Oncol Rep.

