# Supplementary material for: Long non-coding RNA AK027294 involves in the process of proliferation, migration, and apoptosis of colorectal cancer cells
Source: Tumour Biol. 2016 Jan 28;37(8):10097–105. doi: 10.1007/s13277-015-4350-x (PMC4999473; doi:10.1007/s13277-015-4350-x)
Supplement: Supplementary file 2 — (DOCX 160 kb) [file 13277_2015_4350_MOESM2_ESM.docx]

**Figure S2**

**
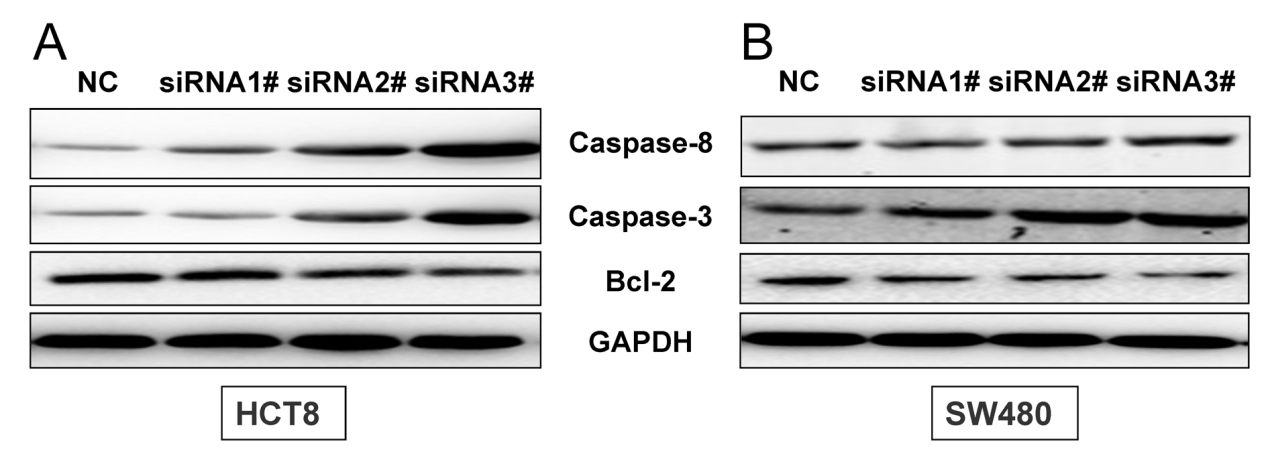
**

Figure S2. The expression of Caspase-8, Caspase-3 and Bcl-2 in HCT8 (A) and SW480 (B) cells treated with negative control siRNA, siRNA1#, siRNA2#, siRNA3# respectively.
